# Window screening, ceilings and closed eaves as sustainable ways to control malaria in Dar es Salaam, Tanzania

**DOI:** 10.1186/1475-2875-8-221

**Published:** 2009-09-29

**Authors:** Sheila B Ogoma, Khadija Kannady, Maggy Sikulu, Prosper P Chaki, Nicodem J Govella, Wolfgang R Mukabana, Gerry F Killeen

**Affiliations:** 1 University of Nairobi, School of Biological Sciences, PO Box 30197-00100 GPO Nairobi, Kenya; 2 Ifakara Health Institute, Coordination Office, PO Box 78373, Kiko Avenue, Mikocheni, Dar es Salaam, United Republic of Tanzania; 3 Dar es Salaam City Council, Ministry of Regional Administration and Local Government, United Republic of Tanzania; 4 Durham University, School of Biological and Biomedical Sciences, South Road, Durham, DH1 3LE, UK; 5 Liverpool School of Tropical Medicine, Vector Group, Pembroke Place, Liverpool, L3 5QA, UK

## Abstract

**Background:**

Malaria transmission in Africa occurs predominantly inside houses where the primary vectors prefer to feed. Human preference and investment in blocking of specific entry points for mosquitoes into houses was evaluated and compared with known entry point preferences of the mosquitoes themselves.

**Methods:**

Cross-sectional household surveys were conducted in urban Dar es Salaam, Tanzania to estimate usage levels of available options for house proofing against mosquito entry, namely window screens, ceilings and blocking of eaves. These surveys also enabled evaluation of household expenditure on screens and ceilings and the motivation behind their installation.

**Results:**

Over three quarters (82.8%) of the 579 houses surveyed in Dar es Salaam had window screens, while almost half (48.9%) had ceilings. Prevention of mosquito entry was cited as a reason for installation of window screens and ceilings by 91.4% (394/431) and 55.7% (127/228) of respondents, respectively, but prevention of malaria was rarely cited (4.3%, 22/508). The median cost of window screens was between US $ 21-30 while that of ceilings was between US $301-400. The market value of insecticide-treated nets, window screening and ceilings currently in use in the city was estimated as 2, 5 and 42 million US$. More than three quarters of the respondents that lacked them said it was too expensive to install ceilings (82.2%) or window screens (75.5%).

**Conclusion:**

High coverage and spending on screens and ceilings implies that these techniques are highly acceptable and excellent uptake can be achieved in urban settings like Dar es Salaam. Effective models for promotion and subsidization should be developed and evaluated, particularly for installation of ceilings that prevent entry via the eaves, which are the most important entry point for mosquitoes that cause malaria, a variety of neglected tropical diseases and the nuisance which motivates uptake.

## Background

Many vector-borne diseases are acquired in the home, usually through exposure to nocturnal, endophilic, and anthropophagic vectors [1]. Fortunately, even very simple changes in house design can protect people against exposure to mosquito bites [2] and malaria infection [3-5]. The primary malaria vectors of Africa prefer feeding on humans in the middle of the night when they are asleep. Thus, they usually have to find their way into the houses to obtain blood and survive [6].

*Anopheles gambiae s.l*. mosquitoes are well adapted for entering houses because they fly upwards when encountering a vertical surface [7]. Attracted by human odour from inside the house they typically reach the wall, travel vertically along its surface and then enter through the eave gap between the wall and the roof. This observation is reinforced by studies showing that houses with open eaves and those lacking ceilings are associated with increased mosquito numbers and higher levels of malaria compared to the ones with closed eaves and the ones with ceilings [3,4]. In The Gambia, children who lived in houses with closed eaves and metal roofs but slept without bed nets had fewer *Plasmodium falciparum *malaria attacks than children who slept in houses with open eaves and also had no bed nets [8].

In the early twentieth century, improved housing and screening were regarded as priority methods of controlling malaria. Italian field experiments on proofing houses against mosquitoes were the very first successful malaria control trials [4,5]. People living in poor houses (incomplete or with walls and roofs made of palm thatch and mud) have a higher exposure to malaria than people occupying houses with complete brick and plaster walls and tile roofs. House screening was also found to reduce mosquito human biting rates as well as malaria infections in settings as diverse as the United States, Greece and Italy [4]. More recently, clinical trials have shown that both full house screening and ceilings alone provide valuable protection against anemia and exposure to malaria transmission in rural parts of The Gambia [9]. The broader potential of window screening, closed eaves and ceilings for preventing entry of a variety of culicine mosquitoes into houses has recently been established in both west and east Africa [[10], Ogoma *et al *unpublished]. Culicines are vectors of a variety of viral and parasitic infections and crucially cause most of the biting nuisance which motivates uptake of household and personal protection measures. The most abundant of these mosquitoes is *Culex quinquefasciatus*, a vector of *Wuchereria bancrofti*, which causes lymphatic filariasis and arboviruses, such as West Nile Virus [11] and Chikungunya [12], mainly in Africa. Others like *Mansonia *sp. transmit *Brugia malayi *and *B. timori *more specifically in south Asian countries [13]. Apart from being disease vectors they are also the most common human-biting culicines, consequently contributing the bulk of nuisance bites, especially in urban areas [14]. The role of nuisance biting mosquitoes in the control of malaria requires particular consideration by control programmes relying upon community participation. For example, low levels of susceptibility of *Cx. quinquefasciatus *to insecticide as was reported in Tanzania [15] and the resulting low efficacy of ITNs against this widespread, nuisance-biting species has been linked to reduced public acceptance of ITNs [16,17].

**Figure 1:**
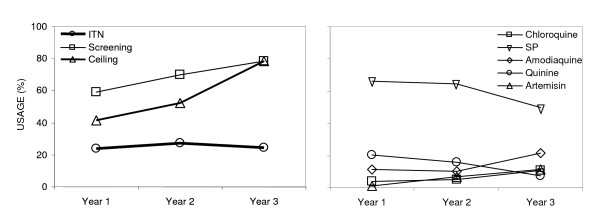
**Time trends of protective measures and drug use in the survey areas of the urban malaria control program**. The overall trends over time were calculated using a logistic regression model with the protection measures and drugs as an outcome. Except for ITN usage (P = 0.507), usage and other protective measures and drugs all significantly increased or decreased (P < 0.001) (Year 1: May 2004-March 2005; Year 2: April 2005-March 2006; Year 3: April 2006- March 2007). Reproduced from reference [18].

This study was carried out in Dar es Salaam, where overall ITN usage has remained consistently and disappointingly low (26% coverage), but window screening and ceiling boards became increasingly common between 2004 and 2006 [18]. Interestingly, this was coupled with a simultaneous decline in malaria prevalence, which could not be explained by changes in coverage of any other intervention (Figure 1). The results of these large, cluster-sampled, cross-sectional surveys, prompted us to further investigate the potential of window screening, ceiling boards and blocking of eaves, particularly considering complementary entomological studies showing that ITNs confer less protection against *An. gambiae *s.s in well-screened and ceilinged houses [18]. Motives behind installation of different types of ceilings and screens by community members were evaluated. Human preference for blocking specific mosquito entry points was compared with the known preference of mosquitoes for various points of entry into the house. In addition, investigations on how much residents spent on ceilings and window screens were done. The overall goal was to understand and improve the acceptability of this intervention for incorporation into integrated vector management strategies.

## Methods

### Study site

This study was carried out in Dar es Salaam, the commercial capital of the Republic of Tanzania, located on the southern coast of the country. The city covers an area of almost 1,400 km^2^, with about 2.7 million inhabitants [19]. It is divided administratively into three municipalities, namely Ilala, Temeke, and Kinondoni. These municipalities are further subdivided into 73 wards. Each ward is divided into several neighborhoods, which are referred to in Kiswahili as *mitaa *(*mtaa *singular). These neighborhoods are divided into ten-cell-units (TCUs) which are the smallest administrative units of local government, headed by an elected leader known as a *Mjumbe*. The TCUs typically comprise of at least 10 to 20 houses, although some may contain even up to 100 houses [20]. Dar es Salaam has a hot and humid tropical climate with two rainy seasons; an intense one during the months of March, April, and May, and a milder one occurring in November and December. The temperatures range between 22°C and 32°C and are typically very suitable for the survival of the primary malaria vectors of Africa, as well as for the development of sporogonic stages of the parasites.

This study was carried out within the study area of the Dar es Salaam Urban Malaria Control Programme (UMCP), which was launched in March 2004. Its main aim was to control aquatic-stage mosquitoes using community based resource persons delivering microbial insecticides through affordable and sustainable implementation systems. The UMCP covers an area of 56 km^2 ^and consists of 15 wards, 67 neighborhoods and more than 3000 TCUs with more than 610,000 residents. The four major activities of the UMCP were application of microbial larvicides, surveillance of aquatic mosquito breeding sites for larvae and pupae, adult mosquito density monitoring and cross-sectional household surveys of individual and household characteristics as well as parasitological assessment of human infection status.

### Sampling design

A total of 150 Ten cell units were randomly sampled (10 TCUs from each ward) from the UMCP study area. All the houses found in each sampled TCU were surveyed between March and August 2008 using a questionnaire, which was designed in English and later translated into *kiSwahili*, the national language. It was pretested for feasibility and clarity and results used to update the questionnaire before the main survey.

The personal interview questionnaire was administered to household heads, or in their absence, the next responsible person, assuming that was an adult of 18 years or above (n = 579 respondents). The survey took place within the home/house of the respondent. Respondents were not prompted with any possible answers during the interview. Information was collected about the condition of the house, including direct observation of the house, the presence or absence of window screens, ceilings and whether the houses had open or closed eaves was recorded (eaves are spaces found between the wall and the roof in a typical traditional African house). The respondents were asked to rank in order of importance, without being presented with a list of alternatives, the reasons for use/installation of different types of window screens and ceilings. Where there was no ceiling and/or screen, they were asked to give their reasons why not. The total reported cost of purchasing screens and/or ceilings was recorded in houses that had them. On the other hand, the total amount of money respondents expected to spend if they were to install screens and ceilings was recorded for those whose houses did not have them. The survey was conducted alongside the Urban Malaria Control Programme (UMCP) household survey via personal interviews. The questionnaire utilized in the household survey was divided into six parts: (i) locational information, (ii) characteristics and structural conditions of the house, (iii) information about the head of the household, (iv) socio-economic and agricultural characteristics of the household, (v) measures for protection against malaria, and (vi) individual, demographic, behavioral and health related information.

### Statistical analysis

The semi-structured part of the questionnaire was coded after completion of the survey. Preliminary analysis and descriptive statistics were processed using Microsoft Excel^®^. All data were entered and analyzed using SPSS 15.0. Analyses of the outcome variables were performed, excluding non-responders or missing data points so the total number of respondents (n) varied between questions. Spearman's Rho-correlation test was used to examine associations between the presence of ceilings, window screens and closed eaves. All pair-wise comparisons between the three variables were tested to examine the association. Partial correlation was also executed in order to establish the relationship between each of the two variables more rigorously by controlling for the effect of the other third variable.

## Results

### Coverage, types and associations between house-proofing methods

The common ways of house proofing recorded in this study area were installation of ceilings, window screens and closed eaves. Over four fifths of the sampled houses in Dar es Salaam had window screens; while almost half had ceilings and only slightly less had closed eaves (Table 1). Crucially, the vast majority (79.8%, 462/579) of the surveyed houses had a ceiling or closed eaves or both (Table 1). This is particularly notable because either method blocks entry into the room space through the eave gap, which is by far the most important entry point for almost all mosquito genera, including *An. gambiae*.

**Table 1:** The proportion of houses with different combinations of mosquito-proofing

**Eaves**	**Ceiling**	**Windows**		**Total**
			
		**Open % (n)**	**Screened % (n)**	
				
Open	Open	2.4% (14)	17.8% (103)	*20.2% (117)*
	Closed	0.8% (5)	37.7% (218)	*38.5% (223)*
	*Subtotal*	*3.2% (19)*	*55.5% (321)*	*58.7% (340)*
				
Closed	Open	12.6% (73)	18.3% (106)	*30.9% (179)*
	Closed	1.4% (8)	9.0% (52)	*10.4% (60)*
	*Subtotal*	*14.0% (81)*	*27.3% (158)*	*41.3% (239)*
				
Total	Open	15.0% (87)	36.0% (209)	51.1% (296)
	Closed	2.0% (13)	48.0% (270)	48.9% (283)
	*Total*	*17.2% (100)*	*82.8% (479)*	*100(579)*

Consistent positive correlations were observed between houses having ceilings, window screens and closed eaves when a bivariate correlation test was applied (Table 2). Partial correlation analyses revealed high correlation in all cases (Table 2), indicating that houses with one of the recorded house-proofing measures were more likely to have one or both of the others.

**Table 2:** Association between different uses of window screening, closed eaves and ceilings

**Condition of the house**	**Screened**		**Closed eaves**	
		
	**r**	**P**	**r**	**P**
				
***Spearmans P test***				
Ceilinged	0.328	< 0.001	0.399	< 0.001
Screened			0.369	< 0.001
***Partial correlation controlling for remaining measures***				
Ceilinged	0.212	<0.001	0.316	<0.001
Screened			0.275	<0.001

**Figure 2:**
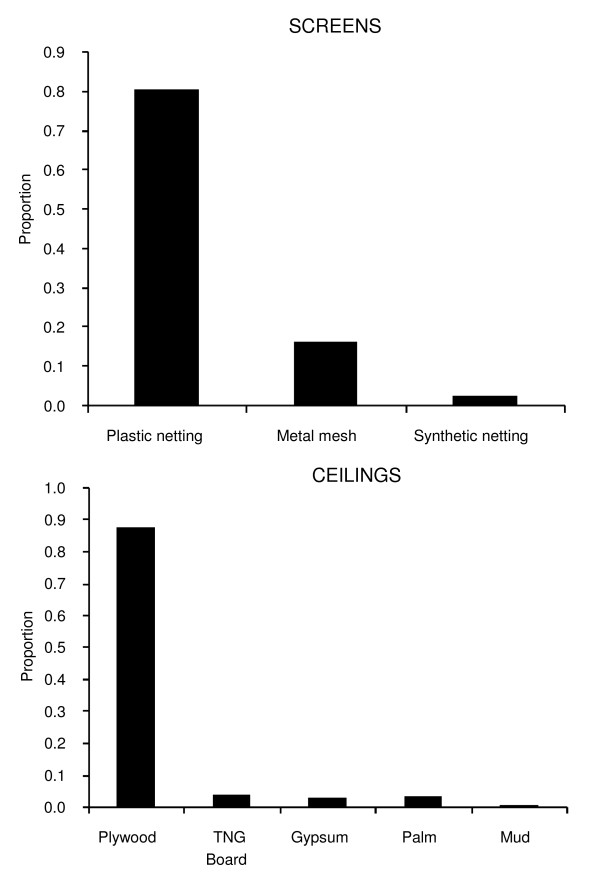
**The proportion of houses with different types of screens and ceilings found in Dar es Salaam city**.

Different types of ceilings were made from the various types of materials available locally. The most common type of ceiling material was plywood board found in almost nine tenths (0.880, 249/283) of sampled houses with ceilings (Figure 2). Other types observed included thin, interlocking wooden panels which are commonly known as "tongue'n groove" (TNG) (0.042, 12/283) and gypsum made from calcium chloride rock (0.032, (9/283) as well as two types of traditional ceiling made from mud (0.011, 3/283) and palm leaves (0.035, 10/283).

More than four fifths of the houses (0.827, 479/579) had screened windows. Of these, the vast majority (0.808, 387/479) was made from plastic netting, while the rest were made from fine metal mesh (0.165, 479/479) and synthetic fiber netting (0.027, 13/479) (Figure 2).

### Motivations and disincentives for installing house-proofing measures

Information about reasons for having a particular type of ceiling was obtained from questions addressed to only those respondents whose houses had ceiling. More than half (55.7%, 127/228) of the respondents cited mosquito entry prevention as a motive. Almost a third (31.6% (72/228)) of respondents cited prevention of entry of mosquitoes as their most important reason and this was the most commonly cited reason (Table 3). This was closely followed by one fifth (21.1%, 48/228) who cited keeping the house cool as their most important reason. Keeping the house cool was also cited by about a quarter of the respondents as the second most important reason, while more than quarter of the respondents said it was more fashionable to install ceilings and cited this as their third most important reason (Table 3). Overall, preventing mosquito entry, keeping the house cool, and current fashion accounted for more than two thirds (68.9%, 350/508) of all reasons cited. Although preventing mosquito entry is of high relevance to public health and was the most common motivation, it is notable that these other two motivations contribute substantially to the desirability of this intervention and most probably played a major role in the high coverage achieved. Prevention of malaria was also cited as a reason for installation but only by a very small proportion of respondents (4.3%, 22/508).

**Table 3:** The proportion of respondents who cited different reasons for installing and/or renting a house with a ceiling

**Reason**	**Importance of reason**				**Total Citations**
		
	**Most**	**Second Most**	**Third Most**	**Fourth Most**	
	**% (n)**	**% (n)**	**% (n)**	**% (n)**	**% (n)**
					
Prevents entry of mosquitoes	31.6 (72)	22.5 (34)	17.1(15)	14.6(6)	25.0 (127)
Keeps the house cool	21.1 (48)	19.8 (30)	21.6 (19)	34.2 (14)	21.9 (111)
Its fashionable	12.3 (28)	28.5 (43)	37.5 (33)	19.5 (8)	22.0 (112)
Its durable	15.4 (35)	15.2 (23)	4.5 (4)	14.6 (6)	13.4 (68)
Prevents entry of dust	3.1 (7)	2.0 (3)	0.0 (0)	0.0 (0)	2.0 (10)
Prevents entry of other insects	0.8 (2)	0.0 (0)	0.0 (0)	0.0 (0)	0.4 (2)
Prevents people from contracting malaria	0.8 (2)	6.0 (9)	8.0 (7)	9.8 (4)	4.3 (22)
Reduces noise from outside	0.0 (0)	0.0 (0)	4.5 (4)	4.9 (2)	1.2 (6)
Its affordable	14.9 (34)	6.0 (9)	6.8 (6)	2.4 (1)	9.8 (50)
**Total**	**100.0 (228)**	**100.0 (151)**	**100.0 (88)**	**100.0 (41)**	**100.0 (508)**

The most commonly cited reason for having window screens was also prevention of entry of mosquitoes but this was much more of a singular, overriding motivation than was the case for ceilings. Almost all (91.4%, 394/431) of the respondents with screens cited this reason as a motive. Almost three quarters of the respondents cited prevention of entry of mosquitoes as their most important reason and this was the most commonly cited reason (Table 4). Other reasons cited included prevention of entry of dust, prevention of noise from outside and prevention of entry of other insects other than mosquitoes (Table 4).

**Table 4:** The proportion of respondents who cited different reasons for installing and or renting houses with window screens

**Reason**	**Importance of Reason**				**Total citations**
		
	**Most**	**Second Most**	**Third Most**	**Fourth Most**	
	**% (n)**	**% (n)**	**% (n)**	**% (n)**	**% (n)**
					
Prevents entry of mosquitoes	72.2 (311)	31.6 (62)	17.9 (19)	4.1 (2)	50.5 (394)
Keeps the house cool	0.2 (1)	10.7 (21)	17.9 (19)	25.0 (12)	6.8 (53)
Its fashionable	0.7 (3)	11.2 (22)	18.9 (20)	18.8 (9)	6.9 (54)
Its durable	10.3 (44)	17.9 (35)	15.1 (16)	18.8 (9)	13.3 (104)
Prevents entry of dust	0.2 (1)	0.5 (1)	0.0 (0)	0.0 (0)	0.3 (2)
Prevents entry of other insects	0.2 (1)	0.0 (0)	0.0 (0)	0.0 (0)	0.1 (1)
Prevents people from contracting malaria	4.9 (21)	16.3 (32)	12.3 (13)	20.8 (10)	9.7 (76)
It enhances security	0.2 (1)	0.0 (0)	0.0 (0)	0.0 (0)	0.1 (1)
Reduces noise from outside	0.0 (0)	0.0 (0)	0.0 (0)	0.0 (0)	0.0 (0)
Its affordable	11.1 (48)	11.8 (23)	17.9 (19)	12.5 (6)	12.3 (96)
**Total**	**100.0 (431)**	**100.0 (196)**	**100.0 (106)**	**100.0 (48)**	**100.0 (781)**

More than three quarters (80.5%, 309/384) of the respondents lacking screens or ceilings considered these to be expensive. A small minority said they had no choice of screens and/or ceilings because they were tenants. Only a small proportion said they did not like having either screens and/or ceilings (Table 5).

**Table 5:** Reasons for lack of screens and ceilings

**Reasons**	**Ceilings**	**Window screens**	**Total citations**
	**% (n)**	**% (n)**	**% (n)**
			
Too expensive	82.2 (235)	75.5 (74)	80.5 (309)
Rented	14.3 (41)	19.4 (19)	15.6 (60)
Don't like	0.7 (2)	1.1 (1)	0.8 (3)
Glass windows	2.5 (7)	2.0 (2)	2.3 (9)
Under construction	0.3 (1)	2.0 (2)	0.8 (3)
Total	100.0 (286)	100.0 (98)	100.0 (384)

### Household expenditure on window screens and ceilings

**Figure 3:**
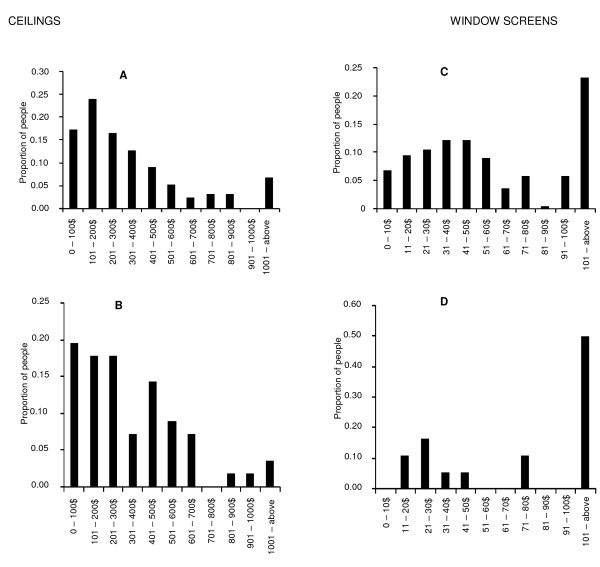
**Graphs showing the cost paid for and expected for ceilings and window screening by respondents**. **A**: The amount of money that has already been spent on the installation of ceilings, **B**; The amount of money that the non-users expected to spend on ceilings, **C**; The amount of money that has already been spent on the installation of window screens, **D**; The amount of money that the nonusers expected to spend on window screens.

Out of 283 (0.489, 283/579) people with houses with ceilings, a quarter (0.470, 133/283) (Figure 3A) mentioned how much they had spent on installation, and a quarter (0.470, 56/296) (Figure 3B) of the nonusers mentioned how much they expected to spend if they were to install them. Out of 479 (0.827, 479/579) people with window screened houses, a quarter (0.457, 219/479) (Figure 3C) mentioned how much they have spent on installation of window screens, while out of 100 nonusers, less than a quarter (0.18, 18/100) (Figure 3) could mention how much they expected to spend, and the rest could not remember how much they spent, or did not know.

The median amount of money that the remainder could remember paying for window screens was US $ 21-30 and ranged from $ 0.9 to 695 (Figure 3). Interestingly, majority of the people who did not have screens expected to pay more than US $ 100. This indicates that most people who lacked screens overestimated the likely cost. In contrast, the amounts of money respondents reported they had paid for, or expected to pay for ceilings, were very similar to each other. Both of these remembered or perceived expenses ranged between US $ 8 and 870, with a median of US $ 301.0 and 400.0 for both the costs incurred and the costs expected (Figure 3).

### Comparison of expenditures upon bed nets, window screens and ceilings

The median number of inhabitants per house was 11. This was used in the calculations of the total expenditure on window screens, ceilings and bed nets. The total expenditure on screens per house was approximately US $ 25 while that of ceilings was US $ 400. Expenditure for window screens per person was almost the same as that of bed nets while for ceilings was 14-fold higher than for bed nets (Table 6). The total amount of money already spent on these three interventions at the time when this study was carried out was calculated and is shown as the total expenditure for the population of the city (Table 6).

**Table 6:** Comparison of total expenditure between different interventions

**Type of intervention**	**Expenditure per person protected**	**Total expenditure for the population of the city**
	**US $**	**US $**
		
Bed nets	2.6^a^	1,831,304
Window screens	2.3^b^	5,182,527
Ceilings	31.9^c^	42,069,559

Expenditure per person = Cost of intervention/Number of people protected.

Expenditure for the city = Cost per person × Population size (The population size used was approximately 2,700,000 inhabitants of Dar es Salaam city × Mean coverage of the intervention.

^a ^The cost of insecticide treated bed nets at the time when this study was carried out was TShs. 6000, at the exchange rate of US $ TShs. 1150 per US $ at the time. Key assumptions are approximately two people per net; ITN coverage was approximately 26%.

^b ^The total median cost of installation of window screens was approximately US $ 25.5 spent on approximately a median of 11 people per house.

^c^The total median cost of installation of ceilings was approximately UD $ 350.5 spent on approximately a median of 11 people per house.

## Discussion

**Figure 4:**
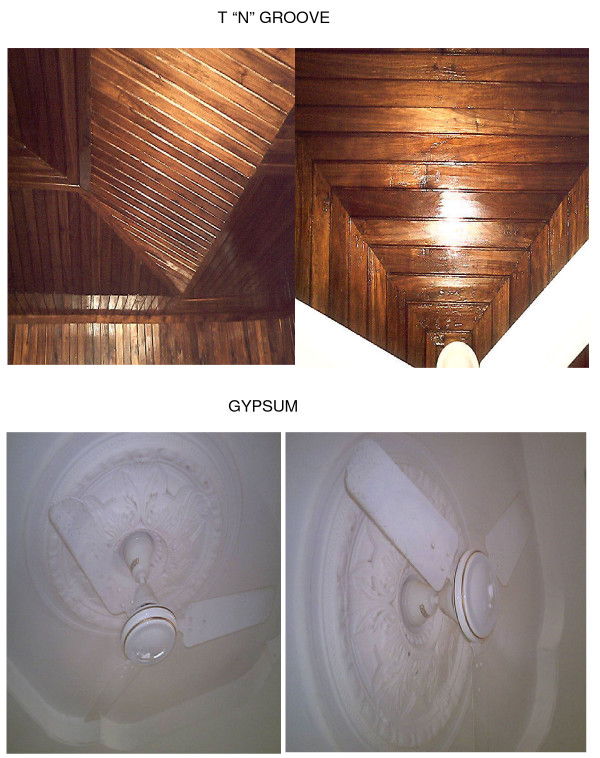
**A photograph of different types of ceilings illustrating the stylish gypsum and tongue and groove types of ceiling**.

Understanding the interactions of the main malaria vectors with changing self-protection behaviors' of humans is essential to success in programmatic settings. In this cross-sectional study, more people lived in houses with screened windows than in houses with ceilings or closed eaves. Nevertheless, the combined coverage of the latter two, both of which prevent house entry by mosquitoes through the eaves, was also high. The general perception of the community was that complete proofing of their houses was more beneficial than partial house proofing, as was illustrated by the positive correlation between ceilings, window screens and closed eaves. Community knowledge of the causative relationship between mosquito bites and malaria transmission was not investigated explicitly. The very low number of respondents who mentioned malaria prevention as a reason for installing any of these measures indicates that their main motivation was to protect against mosquito bites generally. Many residents of Dar es Salaam clearly understood that installation of ceilings protects them from mosquitoes and some even associate this with protection against malaria infection, yet this intervention has not been widely encouraged or promoted. Other motives for installation of ceilings mentioned included fashionability (especially the gypsum type of ceiling which was considered quite stylish; Figure 4) as well as keeping the house cool. This shows that ceilings are perceived to serve more than one function and may be promoted based on multiple benefits. Therefore, it should be relatively easy to promote them, particularly in urban areas where they are probably most appropriate. Indeed, screened ceilings have recently been shown to reduce densities of both the Anophelines that transmit malaria and the Culicines responsible for most of the indoor nuisance biting in both west and east Africa [[3,9,10], Ogoma *et al*., unpublished].

Given the coverage levels observed here, house-proofing may well prove to be a useful strategy for not only equitably protecting entire households against mosquito bites but also for achieving community-level suppression of malaria transmission so that even a remaining minority lacking them benefit from the "mass effect" [21,22]. In fact, in Dar es Salaam this may be already happening: (Figure 1). Steady decline of malaria prevalence between April 2004 and March 2007 was associated with increased coverage of screenings and ceilings while no other intervention experienced substantial increases in coverage except for larviciding which was restricted to only three of the 15 study wards in the final study year (Figure 1) [18].

Generally the residents of the city appeared to try as much as possible to protect themselves against mosquito bites by blocking entry to their houses, as depicted by the rarity of houses (13.8%), which did not have ceilings or windows screens and had open eaves. Perhaps most exciting is the prospect of what might be possible if these materials could be treated with effective, long-lasting insecticide formulations to achieve a substantially enhanced level of household and community-level transmission [21]. This is further substantiated by a study which was carried out in Burkina Faso, West Africa, illustrating substantial reduction in the levels of malaria transmission when permethrin treated curtains were hanged on doors, windows and eaves [23]. Protection of all members within a household, beyond merely those young children and pregnant women at great risk is essential to achieve maximum control and even elimination of malaria [22]. Mosquito-proofing of houses therefore offers the significant advantage of equitably protecting all members of a particular household, even those that are not sleeping under a bed net.

The high coverage of screens and ceilings already attained in Dar es Salaam suggests that this is a vector control measure, which can be readily delivered to large populations in many towns and cities across Africa, particularly if the installment costs can be reduced. This study shows that the initial cost of installing window screens is comparable with that of providing bed nets for all occupants, but more studies should be carried out in order to ascertain the long term cost per person per year based on the durability of these two alternatives. Interestingly most houses which neither had ceilings nor window screens but had closed eaves were initially built this way, and, therefore, no additional cost was required for blocking eaves. Blocking of eaves might well be one the cheapest of the three options but schemes for promoting awareness and understanding of these accessible options for household-based control need to be developed and evaluated. The value of this approach is bolstered by the observation that residents of houses with ceilings, screened windows, and especially the combination of both, take advantage of this protection by spending more time indoors at night [24]. It is particularly striking that while the existing window screening in Dar es Salaam has a greater market value than that of insecticide-treated nets that of ceiling dwarfs either one. With a total market value exceeding US $40 million, this is clearly a tool which the residents of Dar es Salaam have prioritized and invested in and, therefore, has great potential as an intervention tool in and beyond this particular setting.

## Conclusion

Due to high coverage of screens and ceilings, it is concluded that people have readily accepted this method as a way of protecting themselves from mosquito bites and, perhaps inadvertently in many cases, reducing malaria transmission. The prioritization of ceilings to the extent that the residents have paid over US $40 million to install them, suggests ready opportunities for national and international programmes to developing this intervention strategy more proactively and deliberately.

Although cost is the most important constraint on the choice and degree of use of these methods, it is remarkable that coverage with a combination of closed eaves or ceilings equals the 2010 RBM target for ITNs of 80% [25], while that of ITNs in Dar es Salaam remains stagnant at a mere 26% (Figure 1) [18]. This is all the more notable because this particular intervention does not feature in the National Medium Term Strategic Plan and is neither subsidized nor actively promoted.

It seems that most residents of Dar es Salaam know about the value of mosquito-proofing houses but need access to, and information about, cheaper and more durable materials which would ideally have insecticidal and/or excito-repellent properties, which would kill adult mosquitoes directly or act as a more effective barrier for preventing house entry [21]. Moreover, since blocking of eaves seems to be a particularly effective and affordable option, netting materials suitable for window screening could also be used for screening eaves, since they would interfere less with airflow and indoor temperatures than simply blocking this gap.

In order to fully understand the cost-effectiveness of house screening, additional information is required on the durability of the materials used for ceilings and window screens so that the long term effectiveness and costs of this intervention can be determined. In addition, development and evaluation of effective models for promotion and subsidization should be prioritized. Lastly, there is an urgent need to engage policy makers in active consideration of mosquito-proofing houses as one of the tools for integrated control, and perhaps one that can be considered as "low hanging fruit" in the urban context.

## Competing interests

The authors declare that they have no competing interests.

## Authors' contributions

SBO designed the questionnaire used in the study, participated in the collection of data, analyzed the data and drafted the manuscript in consultation with the other authors. KK, implemented and managed the Urban Malaria Control Program in which this study was undertaken, including the household surveys, assisted in development of the questionnaire, and assisted in drafting the manuscript. MS, PPC, and NJG helped supervise collection of the data and drafting of the manuscript. WRM supervised the study and participated in drafting the manuscript; GFK conceived the study and supervised all aspects of the experimental design, data analysis, interpretation of data and drafting of the manuscript. All authors read and approved the final manuscript.
